# Telemedicine utilization and digital inclusion among people with disabilities in Saudi Arabia: post-pandemic associated factors and policy implications

**DOI:** 10.3389/fpubh.2026.1756958

**Published:** 2026-05-11

**Authors:** Osama Albasheer, Siddig Ibrahim Abdelwahab, Ibrahim Gosadi, Hatim Alessa, Afnan Madkhali, Afaf Hakami, Hanin Jaber Mobarki, Osama Ahmed Mobarki, Jawaher Sultan Farji, Rahf Ali Hakami, Khalid Mohammed Aldalgan, Murad Ahmed Mobarki, Atheer Abdulhadi Homadi, Najla Abdulrahman Alhazmi, Alhussain Mohammed Hakami

**Affiliations:** 1 Department of Family and Community Medicine, Faculty of Medicine, Jazan University, Jazan, Saudi Arabia; 2 Health Research Centre, Jazan University, Jazan, Saudi Arabia; 3 Department of Family Medicine, Jazan University Hospital, Jazan University, Jazan, Saudi Arabia; 4 Faculty of Medicine, Jazan University, Jazan, Saudi Arabia

**Keywords:** digital health, disability evaluation, disabled individuals, health services accessibility, Saudi Arabia, telemedicine

## Abstract

**Background:**

People with disabilities face substantial barriers to accessing healthcare services, particularly in regions with limited physical and digital infrastructure. This study aimed to assess the prevalence of telemedicine utilization among individuals with disabilities in Saudi Arabia, identify key associated factors of its adoption, and explore the main barriers to its use.

**Method:**

A cross-sectional survey was conducted between December 2024 and April 2025 among 488 individuals with disabilities from Saudi Arabia. Participants were assessed using the World Health Organization Disability Assessment Schedule 2.0 (WHODAS 2.0) for functional limitations and the Telehealth Usability Questionnaire (TUQ-21) to evaluate telemedicine experience and satisfaction. Data were collected through face-to-face interviews conducted by trained healthcare professionals. Descriptive statistics, chi-square tests, and multivariate logistic regression analyses were used to examine factors associated with telemedicine use.

**Results:**

Among the 488 participants, only 25.4% (*n* = 124) reported using telemedicine service. WHODAS scores were significantly higher among individuals who did not use telemedicine (mean ± SD: 2.55 ± 1.08) compared to users (mean ± SD: 2.49 ± 1.00). Higher education levels and urban residence were significantly associated with telemedicine utilization, while being widowed was associated with lower use. Psychological conditions showed the strongest association with telemedicine use (*χ*^2^ = 45.915, *p* < 0.001). Major barriers included a preference for in-person visits (81.9%), lack of familiarity with telehealth (27.2%), and limited provider availability (22.0%).

**Conclusion:**

Despite favorable perceptions of privacy and ease of use, telemedicine adoption among people with disabilities in Saudi Arabia remains low. Enhancing digital literacy, provider capacity, and service availability is critical to expanding access.

## 1 Introduction

A disability is a long-term or permanent physical, mental, intellectual, or sensory impairment that limits individuals from fully and equitably participating in society (1). People with disabilities, particularly in rural or underserved areas, often require both general and specialized healthcare services but face barriers due to physical, economic, technological, and social limitations (2–4).

Digital transformation, the integration of digital technologies into all aspects of healthcare systems, has reshaped service delivery and improved operational resilience, particularly during and after the COVID-19 pandemic (5–7). In Saudi Arabia, the digital health ecosystem has rapidly evolved, driven by Vision 2030 goals and supported by national platforms such as Seha Virtual Hospital, which now spans over 200 hospitals and offers 44 specialties (8). These developments highlight the nation’s commitment to expanding virtual healthcare access and improving patient outcomes through innovation (8–11).

Telemedicine offers significant promise for addressing access challenges among individuals with disabilities by enabling remote consultations, monitoring, and therapeutic services (5). For example, virtual physical therapy, psychiatric tele-counseling, and assistive digital tools for communication have demonstrated benefit for individuals with mobility, sensory, or cognitive impairments (6, 7, 12). A growing demand for telenursing and remote services among older adults with disabilities in Saudi Arabia further supports the need for inclusive telehealth policies (12).

Despite these advances, the adoption of telemedicine among individuals with disabilities remains underexplored in Saudi Arabia. While some studies have addressed public awareness (13), and provider perceptions of telehealth implementation (14), there remains a significant gap in empirical evidence regarding utilization, associated factors, and barriers specific to the disabled population. Moreover, ethical and legal frameworks around tele-rehabilitation are still evolving and may influence user trust and provider engagement (15).

This study aims to assess the prevalence of telemedicine use among individuals with disabilities in Saudi Arabia and examine the association between disability severity and telemedicine utilization. Additionally, it seeks to identify sociodemographic factors that influence telemedicine adoption and evaluate user perceptions and satisfaction with telemedicine services.

Finally, the study highlights key barriers and facilitators to telemedicine adoption among people with disabilities, contributing to the ongoing discourse on digital health inclusion and informing future policies aimed at enhancing telemedicine accessibility for marginalized populations in Saudi Arabia and beyond. Given the national focus on digital healthcare expansion, the findings of this study will provide critical insights for policymakers, healthcare providers, and technology developers to optimize telemedicine services for individuals with disabilities, ensuring equitable and inclusive healthcare access.

## 2 Materials and methods

### 2.1 Study design and setting

The current study employed a cross-sectional survey design and was conducted across both urban and rural regions to ensure diverse representation. Data were collected from December 10, 2024, to April 21, 2025, using structured questionnaires and standardized assessment tools.

### 2.2 Study population

#### 2.2.1 Inclusion criteria

The target population included Saudi individuals aged 18 years or older with any type of disability living in urban or rural areas who had presented for healthcare services at selected primary, secondary, or tertiary healthcare facilities. Additionally, parents of children with disabilities, regardless of the child’s age, were included to capture a broader spectrum of disability-related healthcare experiences.

#### 2.2.2 Exclusion criteria

Those individuals who were unable to communicate owing to cognitive or severe sensory impairments, patients who were hospitalized, or those who presented for reasons that were not connected to disability were not included in the study of participants. Additionally, telemedicine use was not required for participation, as the study aimed to assess its prevalence among individuals with disabilities rather than limit inclusion to prior users.

### 2.3 Sample size and the sampling technique

The sample size was calculated using Epi Info software, based on a 95% confidence level, 5% margin of error, and an expected prevalence of 50% for telemedicine use. In the absence of prior population-based estimates of telemedicine utilization among people with disabilities in Saudi Arabia, an expected prevalence of 50% was adopted as a conservative epidemiological assumption. This approach is commonly recommended when baseline prevalence is unknown, as it ensures sufficient sample size, maintains statistical precision, and minimizes the risk of underpowered subgroup and multivariable analyses (16). The minimum required sample size was 384 participants. To account for potential non-responses and incomplete data, an additional 10% was added, resulting in a target sample of 422. To further enhance representativeness and data completeness, the final sample included 488 individuals.

A stratified random sampling technique was employed to ensure proportional representation of both urban and rural populations. Participants were recruited from various healthcare settings including primary, secondary, and tertiary care facilities across multiple regions in Saudi Arabia.

### 2.4 Data collection tools and measures

Data were collected through direct face-to-face interviews conducted by trained medical students. All interviewers underwent structured training in mobile health (mHealth) principles, standardized data collection procedures, and research ethics during a 2-day workshop facilitated by the research team. The training included practical sessions on administering structured questionnaires, employing effective communication strategies with participants with disabilities, and ensuring data quality, accuracy, and participant confidentiality. This approach guaranteed a substantial response rate, reduced misinterpretations, and offered a chance to elucidate any unclear inquiries for individuals with impairments. Utilizing skilled experts for conducting interviews facilitated the inclusion of persons with sensory, cognitive, or communication impairments, hence promoting inclusiveness in data gathering.

The questionnaire was composed of standardized, validated tools. Demographic and socioeconomic information like; Age, gender, marital status, education level, work status, and living situation, residence, and disability type and severity were collected first.

Disability types were categorized based on participants’ self-reports into physical, sensory (visual or hearing), neurological, psychological, and emotional domains. Psychological conditions referred to self-reported, clinician-diagnosed mental health disorders such as depression or anxiety. In contrast, emotional difficulties captured subjective emotional distress (e.g., persistent stress, sadness, or emotional instability) that did not necessarily correspond to a formal psychiatric diagnosis. These categories were not mutually exclusive.

Functioning and limitations were assessed via the World Health Organization Disability Assessment Schedule 2.0 (WHODAS 2.0) (17), which is available in different languages. An Arabic version of the questionnaire was used after an official permission via the email. The questionnaire covers 6 domains: cognitive, mobility, self-care, interacting with other people, life activities and participation in community activities.

Usability and perceptions of telemedicine were assessed using the Telehealth Usability Questionnaire (TUQ-21), a validated instrument developed by Parmanto et al. (18). The validated Arabic version of TUQ-21 was generated and utilized with appropriate authorization in previous studies (19, 20). TUQ comprises 21 items across six subscales: usefulness (3 items), ease of use (3 items), reliability (3 items), interface quality (4 items), interaction quality (4 items), and satisfaction and future use (4 items) (18). The instrument employs a five-point Likert scale ranging from (1) “strongly disagree” to (5) “strongly agree.” The analysis of usability items, subscale scores, and TUQ scores was conducted using weighted mean scores, categorized as follows: strongly disagree (1.00–1.80), disagree (1.81–2.60), neutral (2.61–3.40), agree (3.41–4.20), and strongly agree (4.21–5.00). The categorization was divided into three levels: low (≤3.39), moderate (3.40–3.79), and high (≥3.80) to assess the perceived usability of telehealth.

A pilot study involving 30 participants was conducted to test the questionnaire’s clarity and reliability. Based on the panel feedback, minor adjustments were made to enhance comprehension and question relevance. The pilot responses were excluded from the main analysis. Reliability was confirmed using the test–retest method, yielding a Cronbach’s alpha of 0.78, indicating good internal consistency (21).

Participants were identified and approached at outpatient clinics within primary, secondary, and tertiary care facilities. Eligible individuals were screened based on inclusion criteria by trained medical students. Potential participants were informed about the study purpose and invited to participate voluntarily. Written informed consent was obtained prior to data collection. This process was designed to ensure inclusivity and minimize selection bias.

### 2.5 Statistical analysis

Statistical analyses were performed using SPSS software (version 22). Due to the use of face-to-face interviews and real-time data validation by trained healthcare staff, the dataset had no missing values, and all analyses were conducted on complete cases. Descriptive statistics (frequencies, means, and standard deviations) were computed for all variables. WHODAS scores were analyzed to assess the severity of disability. Chi-square tests were conducted to determine associations between telemedicine use and categorical variables (e.g., marital status, education level, and employment status). Multicollinearity was assessed before model fitting. Tests for barriers to telemedicine use were performed to examine significant factors influencing non-utilization. Multivariate logistic regression analysis was used to identify associated factors of telemedicine adoption, adjusting for education, residence, and disability type, results were reported using adjusted odds ratios (AORs) with 95% confidence intervals (CIs) provided for regression results. *p*-values < 0.05 were considered statistically significant.

### 2.6 Institutional review board statement

Ethical approval was obtained from the Jazan Health Cluster Ethics Committee, Saudi Arabia (No. 2487 on 02/12/2024). This study adhered to ethical standards within the geographical boundaries of the Kingdom of Saudi Arabia. Participants’ anonymity was prioritized, and strict confidentiality was maintained for all collected questionnaires.

## 3 Results

### 3.1 Association between demographic characteristics and WHODAS score among individuals with disabilities

Table 1 summarizes the key demographic characteristics of the study participants. The majority were aged 40–65 years (38.3%) and female (68.6%). Educational attainment varied, with 39.3% completing school or pre-university education, 35.5% being illiterate, and 23.2% holding university degrees. Most participants were married (43%) or never married (33%), and the most common employment status was housewife (39.3%). Nearly all respondents lived with family (94.3%), and residency was almost equally split between urban (50.6%) and rural (49.4%) areas. Regarding telehealth use, 25.4% used telemedicine services in the past year, while 74.6% did not. Significant variations in WHODAS 2.0 scores were observed across age (*p* = 0.00), education (*p* = 0.00), marital status (*p* = 0.00), work status (*p* = 0.00), and living situation (*p* = 0.00). Higher disability scores were reported among older adults (≥65 years: 3.20 ± 1.13), illiterate individuals (2.88 ± 1.12), widowed participants, and retirees. In contrast, university graduates (2.06 ± 0.81) and those aged 18–24 years (1.83 ± 0.71) had the lowest scores. No significant differences were found based on gender or residence. Additionally, mean WHODAS scores were slightly higher among non-users of telemedicine compared with users (2.55 vs. 2.49); however, this difference was not statistically significant (*p* = 0.545).

**Table 1:** Association between demographic characteristics and WHODAS score among individuals with disabilities.

WHODAS score					
Variables	Categories	*N*	%	Mean ± SD	*p*-value
Age	Less than 18	66	13.50	2.47 ± 1.16	0.000
	18–24 years	23	4.70	1.83 ± 0.71	
	24–39 years	129	26.40	2.26 ± 0.98	
	40–65 years	187	38.30	2.54 ± 0.94	
	More than 65 years	83	17.00	3.2 ± 1.13	
Gender	Male	153	31.40	2.51 ± 1.08	0.681
	Female	335	68.60	2.55 ± 1.05	
Education	Illiterate	173	35.50	2.88 ± 1.12	0.000
	School or pre-university	192	39.30	2.52 ± 1.03	
	University	113	23.20	2.06 ± 0.81	
	Post-graduate	10	2.00	2.52 ± 1.17	
Marital status	Never married	161	33.00	2.35 ± 1.11	0.000
	Currently married	210	43.00	2.46 ± 0.97	
	Separated	28	5.70	2.54 ± 0.94	
	Divorced	15	3.10	2.5 ± 0.94	
	Widowed	74	15.20	3.2 ± 1.06	
Work status	Paid work	72	14.80	2.06 ± 0.86	0.000
	Unemployed for health reasons	22	4.50	2.67 ± 1.04	
	Student	72	14.80	2.38 ± 1.14	
	Retired	66	13.50	2.76 ± 1.05	
	Unemployed for other reasons	45	9.20	2.44 ± 1.04	
	Housewife	192	39.30	2.77 ± 1.05	
	Self-employed	19	3.90	2.0 ± 0.84	
Residence	Rural	241	49.40	2.54 ± 1.02	0.969
	Urban	247	50.60	2.54 ± 1.11	
Living situation	Alone	26	5.30	2.08 ± 1.0	0.068
	With family	460	94.30	2.57 ± 1.06	
	Institutional care	1	0.2		
	Other	1	0.2		
Use of telemedicine	No	364	74.6	2.55 ± 1.08	0.545
	Yes	124	25.4	2.49 ± 1.0	
Total		488	100	2.54 ± 1.06	

### 3.2 Disability categories among the study participants

**Figure 1:**
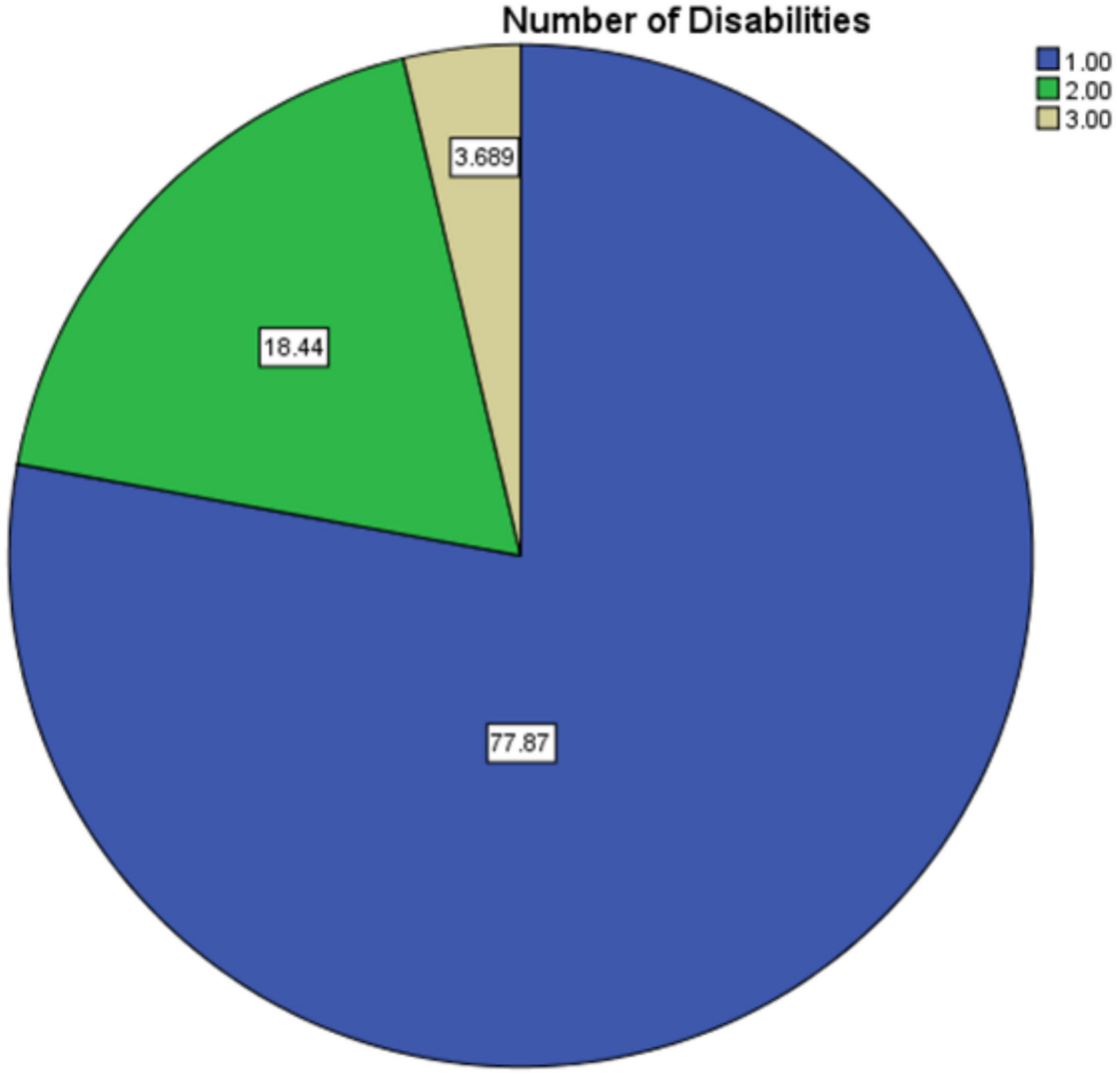
Distribution of multiple disabilities (%) among participants based on self-reported primary and secondary disability types as recorded during data collection.

Figure 1 provides a summary of the distribution of participants according to the presence of different impairments. In the survey, 77.9% of persons (*n* = 380) reported having just one kind of impairment, whereas 18.4% of individuals (*n* = 90) reported having two different types of disabilities. The percentage of people who suffered three or more impairments was low, coming in at 3.7% (*n* = 18). The findings of this study suggest that while the majority of the persons in the sample had a single handicap, a sizeable number of them suffered multiple disabilities. This may have further consequences for the accessibility issues they face and the healthcare requirements they have. According to the cumulative percentages, 96.3% of the participants had one or two impairments, and just a tiny minority of the participants (3.7%) had three disabilities (Figure 1).

The WHODAS 2.0 mean scores and standard deviations for a variety of functional limitations are shown in Table 2. Additionally, the frequency and percentage distribution of these difficulties across severity levels is also included. With 27.7% of participants reporting severe difficulty, the difficulty of standing for extended periods of time was discovered to have the highest mean score (3.13 ± 1.57). This was followed by the difficulty of engaging in social activities (3.06 ± 1.60) and the difficulty of moving about independently (3.03 ± 1.54). In contrast, the mean scores for difficulties in following instructions (1.80 ± 1.28) and handling personal finances (1.85 ± 1.28) were the lowest. The majority of participants had no problem in either of these areas, with 63.9 and 61.9% of them, respectively, saying that they had no trouble. The mean scores for difficulties in establishing new friends or engaging (2.03 ± 1.39) and challenges with everyday activities due to health (2.01 ± 1.36) were likewise lower. However, more than half of the respondents did not experience any difficulty in any of these areas (56.6 and 55.5%, respectively). Particularly noteworthy is the fact that difficulties in understanding or communicating (2.94 ± 1.51) and problems with emotional wellbeing as a result of health (2.63 ± 1.54) demonstrated a reasonably high percentage of severe or extreme instances. The results of this study suggest that there is a significant amount of variation in functional impairments, with mobility and social involvement appearing as the most significant issues among the participants (Table 2).

**Table 2:** The WHODAS 2.0 scores across different domains of daily life among individuals with disability and functional impairments.

WHODAS items	Mean ± SD	*N* (%)				
		None	Mild	Moderate	Severe	Extreme
Difficulty standing for long periods.	3.13 ± 1.57	127 (26.0)	58 (11.9)	63 (12.9)	105 (21.5)	135 (27.7)
Challenges in getting around on your own.	3.03 ± 1.54	128 (26.2)	61 (12.5)	90 (18.4)	84 (17.2)	125 (25.6)
Trouble managing personal hygiene (e.g., bathing).	2.87 ± 1.53	137 (28.1)	83 (17.0)	84 (17.2)	73 (15.0)	111 (22.7)
Difficulty understanding or communicating.	2.94 ± 1.51	127 (26.0)	78 (16.0)	92 (18.9)	79 (16.2)	112 (23.0)
Challenges with day-to-day responsibilities.	2.60 ± 1.48	172 (35.2)	77 (15.8)	96 (19.7)	62 (12.7)	81 (16.6)
Problems with learning or concentrating.	2.55 ± 1.45	176 (36.1)	81 (16.6)	83 (17.0)	83 (17.0)	65 (13.3)
Trouble participating in social activities.	3.06 ± 1.60	135 (27.7)	63 (12.9)	69 (14.1)	78 (16.0)	143 (29.3)
Difficulty making new friends or interacting.	2.03 ± 1.39	276 (56.6)	60 (12.3)	67 (13.7)	32 (6.6)	53 (10.9)
Problems with daily activities due to health.	2.01 ± 1.36	271 (55.5)	71 (14.5)	66 (13.5)	32 (6.6)	48 (9.8)
Struggles managing personal finances.	1.85 ± 1.28	302 (61.9)	60 (12.3)	64 (13.1)	22 (4.5)	40 (8.2)
Difficulty following directions.	1.80 ± 1.28	312 (63.9)	62 (12.7)	54 (11.1)	19 (3.9)	41 (8.4)
Issues with emotional wellbeing due to health.	2.63 ± 1.54	185 (37.9)	61 (12.5)	82 (16.8)	72 (14.8)	88 (18.0)

### 3.3 Accessibility and perceived understanding in healthcare for individuals with disabilities

**Figure 2:**
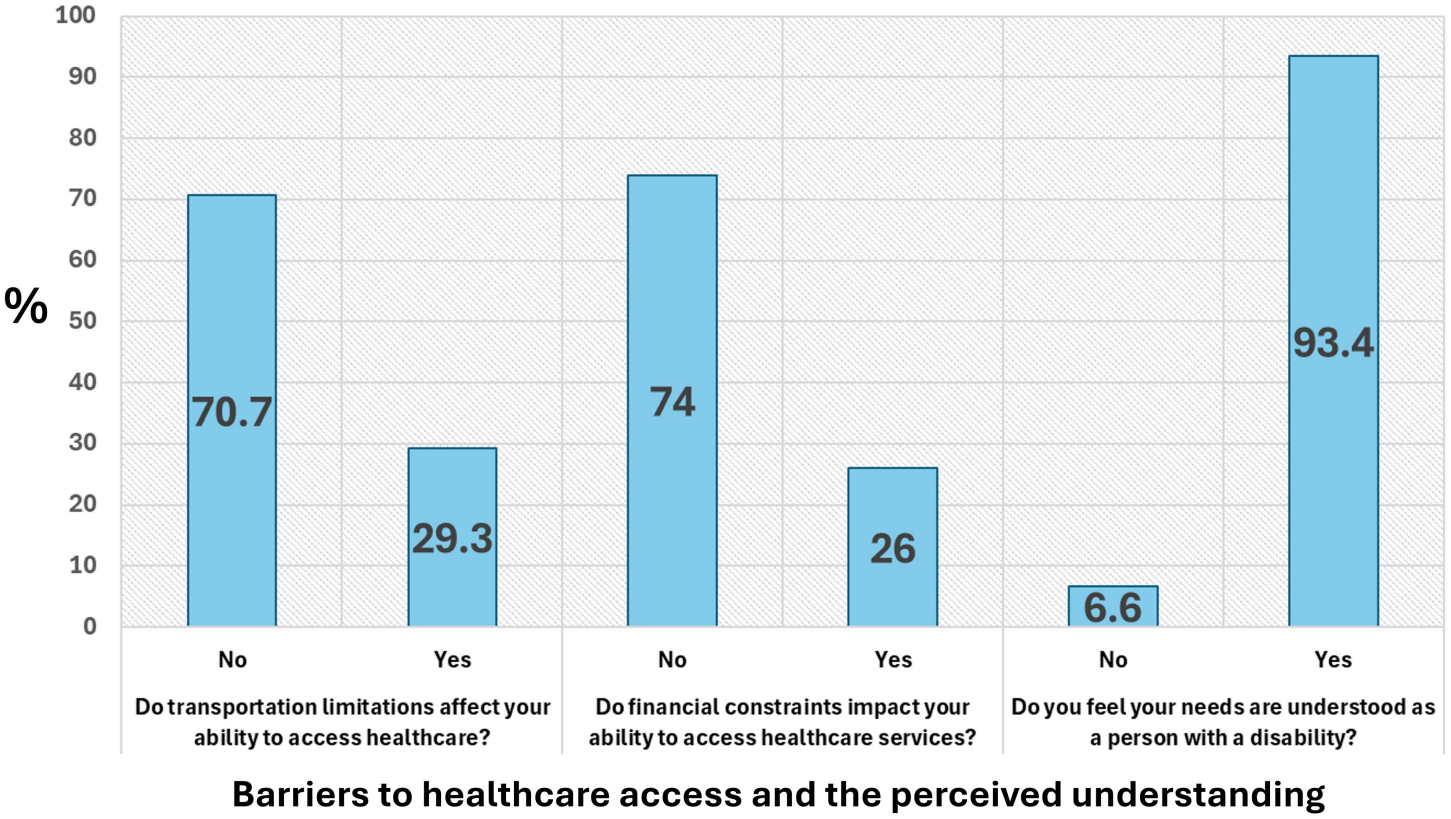
Participants’ responses regarding key barriers to healthcare access and the perceived understanding of their needs, along with the results of chi-square tests.

Figure 2 summarizes participants’ responses regarding key barriers to healthcare access and the perceived understanding of their needs, along with the results of chi-square tests. A majority of participants (70.7%) reported no transportation difficulties, while 29.3% experienced such limitations, with a significant association identified (*χ*^2^(1) = 83.615, *p* < 0.001). Similarly, 74.0% reported no financial constraints, whereas 26.0% did, and this relationship was also statistically significant (*χ*^2^(1) = 112.205, *p* < 0.001). Notably, 93.4% of participants felt that their needs as persons with disabilities were understood, with only 6.6% reporting otherwise (*χ*^2^(1) = 368.393, *p* < 0.001). These findings highlight that while the majority felt well-understood in their healthcare interactions, transportation and financial challenges remain significant barriers for a notable portion of the population.

### 3.4 Experience and use of telemedicine based on telemedicine (TUQ-21 items) scale

Table 3 provides a summary of the descriptive data of the use of telemedicine (TUQ-21 Items) scale, which is used to evaluate user perceptions across a variety of characteristics of telemedicine services. The standard deviations for the 21 items varied from 0.81 to 1.06, while the mean scores for the items ranged from 3.94 to 4.38. This indicates that the majority of people had positive experiences and positive impressions of telemedicine. With a mean score of 4.38 ± 0.81, the item “The telemedicine platform feels secure and private” had the highest mean score. There were 52.4% of respondents who strongly agreed with this statement. In a similar line, the item “I trust that my personal health information is protected during telemedicine sessions” obtained a high level of agreement, with a mean score of 4.32 ± 0.86. Items such as “The telemedicine system is easy to use” and “I can easily learn how to use the telemedicine platform” also received excellent ratings in terms of usability. The mean scores for these items were 4.21 ± 1.01 and 4.17 ± 1.03, respectively. The measures that were used to evaluate satisfaction and dependability, such as “Telemedicine services work without frequent interruptions” (4.07 ± 0.96) and “I am satisfied with my experience using telemedicine” (4.06 ± 0.93), consistently received good replies from the respondents. It is important to note that there was the maximum degree of agreement about matters pertaining to privacy and security. On the other hand, satisfaction and quality of interaction earned somewhat lower marks, despite the fact that they were still positive. An overall good appraisal of telemedicine services was seen among the participants, as indicated by the TUQ-21 Score, which was reported to be 4.14 ± 0.78. The sample size for this study was stated to be 124. These findings underline the fact that participants usually perceived telemedicine to be secure, simple to use, and helpful in satisfying their healthcare requirements. This reflects favourable overall impressions of telemedicine services.

**Table 3:** Descriptive statistics of the use of telemedicine (TUQ-21 items) scale.

Descriptive statistics	*N* (%)						
Items	*N*	Mean ± SD	Strongly disagree	Disagree	Neutral	Agree	Strongly agree
Use-telemedicine allows me to receive healthcare when I otherwise could not.	123	4.22 ± 0.89	3 (2.4)	3 (2.4)	11 (8.9)	53 (43.1)	53 (43.1)
Use-Telemedicine helps me manage my health more effectively.	124	4.17 ± 0.98	5 (4.0)	4 (3.2)	8 (6.5)	55 (44.4)	52 (41.9)
Use-Telemedicine saves me time compared to attending in-person visits.	124	4.19 ± 0.97	4 (3.2)	3 (2.4)	15 (12.1)	45 (36.3)	57 (46.0)
Use-Using telemedicine makes it easier for me to communicate with healthcare providers.	124	4.10 ± 1.05	7 (5.6)	2 (1.6)	14 (11.3)	50 (40.3)	51 (41.1)
Ease-The telemedicine system is easy to use.	124	4.21 ± 1.01	4 (3.2)	6 (4.8)	11 (8.9)	42 (33.9)	61 (49.2)
Ease-I can easily learn how to use the telemedicine platform.	124	4.17 ± 1.03	4 (3.2)	6 (4.8)	14 (11.3)	41 (33.1)	59 (47.6)
Ease-I can complete my healthcare tasks efficiently using telemedicine.	124	4.10 ± 1.03	4 (3.2)	7 (5.6)	15 (12.1)	44 (35.5)	54 (43.5)
Ease-The telemedicine platform is simple to navigate.	124	4.18 ± 1.02	5 (4.0)	5 (4.0)	10 (8.1)	47 (37.9)	57 (46.0)
Interaction-I feel comfortable communicating with healthcare providers using telemedicine.	124	4.10 ± 1.02	3 (2.4)	5 (4.0)	22 (17.7)	41 (33.1)	53 (42.7)
Interaction-The quality of communication with my healthcare provider through telemedicine is good.	124	4.10 ± 0.99	5 (4.0)	6 (4.8)	19 (15.3)	43 (34.7)	51 (41.1)
Interaction-My healthcare provider understands my health needs during telemedicine consultations.	124	4.04 ± 1.06	3 (2.4)	1 (0.8)	15 (12.1)	52 (41.9)	53 (42.7)
Reliability-The audio and video quality of telemedicine sessions is reliable.	124	4.22 ± 0.87	3 (2.4)	4 (3.2)	23 (18.5)	45 (36.3)	49 (39.5)
Reliability-telemedicine services work without frequent interruptions.	124	4.07 ± 0.96	3 (2.4)	8 (6.5)	15 (12.1)	45 (36.3)	53 (42.7)
Reliability-The telemedicine platform is available when I need it.	124	4.10 ± 1.01	3 (2.4)	5 (4.0)	16 (12.9)	57 (46.0)	43 (34.7)
Satisfaction-I am satisfied with my experience using telemedicine.	124	4.06 ± 0.93	4 (3.2)	4 (3.2)	23 (18.5)	53 (42.7)	40 (32.3)
Satisfaction-Telemedicine meets my healthcare needs.	124	3.98 ± 0.97	5 (4.0)	3 (2.4)	24 (19.4)	47 (37.9)	45 (36.3)
Satisfaction-I would use telemedicine again in the future.	124	4.00 ± 1.01	6 (4.8)	5 (4.0)	22 (17.7)	48 (38.7)	43 (34.7)
Satisfaction-I would recommend telemedicine to others.	124	3.94 ± 1.06	2 (1.6)	1 (0.8)	11 (8.9)	44 (35.5)	66 (53.2)
Privacy-The telemedicine platform feels secure and private.	124	4.38 ± 0.81	2 (1.6)	1 (0.8)	17 (13.7)	39 (31.5)	65 (52.4)
Privacy-I trust that my personal health information is protected during telemedicine sessions.	124	4.32 ± 0.86	2 (1.6)	2 (1.6)	12 (9.7)	43 (34.7)	65 (52.4)
Privacy-I feel confident sharing my health information through telemedicine.	124	4.35 ± 0.85	3 (2.4)	3 (2.4)	11 (8.9)	53 (43.1)	53 (43.1)
Score	4.14 ± 0.78 (N = 124)						

The cross-tabulation and chi-square test findings for the use of telemedicine are shown in Table 4, which is organized according to the various health problems. According to the findings of the research, there is a significant correlation between the use of telemedicine and the presence of physical impairments (*χ*^2^ = 5.358, *p* = 0.021). Specifically, 4.9% of persons with physical disabilities utilized telemedicine, whereas 25.8% of individuals did not utilize telemedicine. Visual sensory (*p* = 0.202), hearing sensory (*p* = 0.341), neurological (*p* = 0.686), and emotional conditions (*p* = 0.617) were found to have no significant relationships with the use of telemedicine. 9.2% of patients who were diagnosed with psychiatric problems used telemedicine, whereas 88% did not. This indicates a high and significant connection with the utilization of telemedicine (χ2 = 45.915, *p* < 0.001). Furthermore, 11.1% of those who suffered from emotional problems used telemedicine, while 88.5% did not, even though this specific link did not meet the criteria for statistical significance. Based on these data, it seems that problems that are physical (30.7%) and psychological (97.3%) play a major role in the adoption of telemedicine, but other health conditions do not demonstrate any significant connections with the use of telemedicine.

**Table 4:** Association between different types of disabilities and the utilization of telemedicine services, using the Pearson chi-square test.

Category	Telemedicine *N* (%)			Pearson chi-square	*p*-value
	No	Yes	Total count (%)		
Physical	126 (25.8%)	24 (4.9%)	150 (30.7%)	5.358	0.021
Visual sensory	309 (63.4%)	34 (7.0%)	343 (70.4%)	1.627	0.202
Hearing sensory	355 (72.7%)	47 (9.6%)	402 (82.4%)	0.908	0.341
Neurological	407 (83.6%)	50 (10.3%)	457 (93.8%)	0.163	0.686
Psychological	430 (88.1%)	45 (9.2%)	475 (97.3%)	45.915	<0.001
Emotional	432 (88.5%)	54 (11.1%)	486 (99.6%)	0.250	0.617

### 3.5 Barriers and challenges of telemedicine utilization among non-users

**Figure 3:**
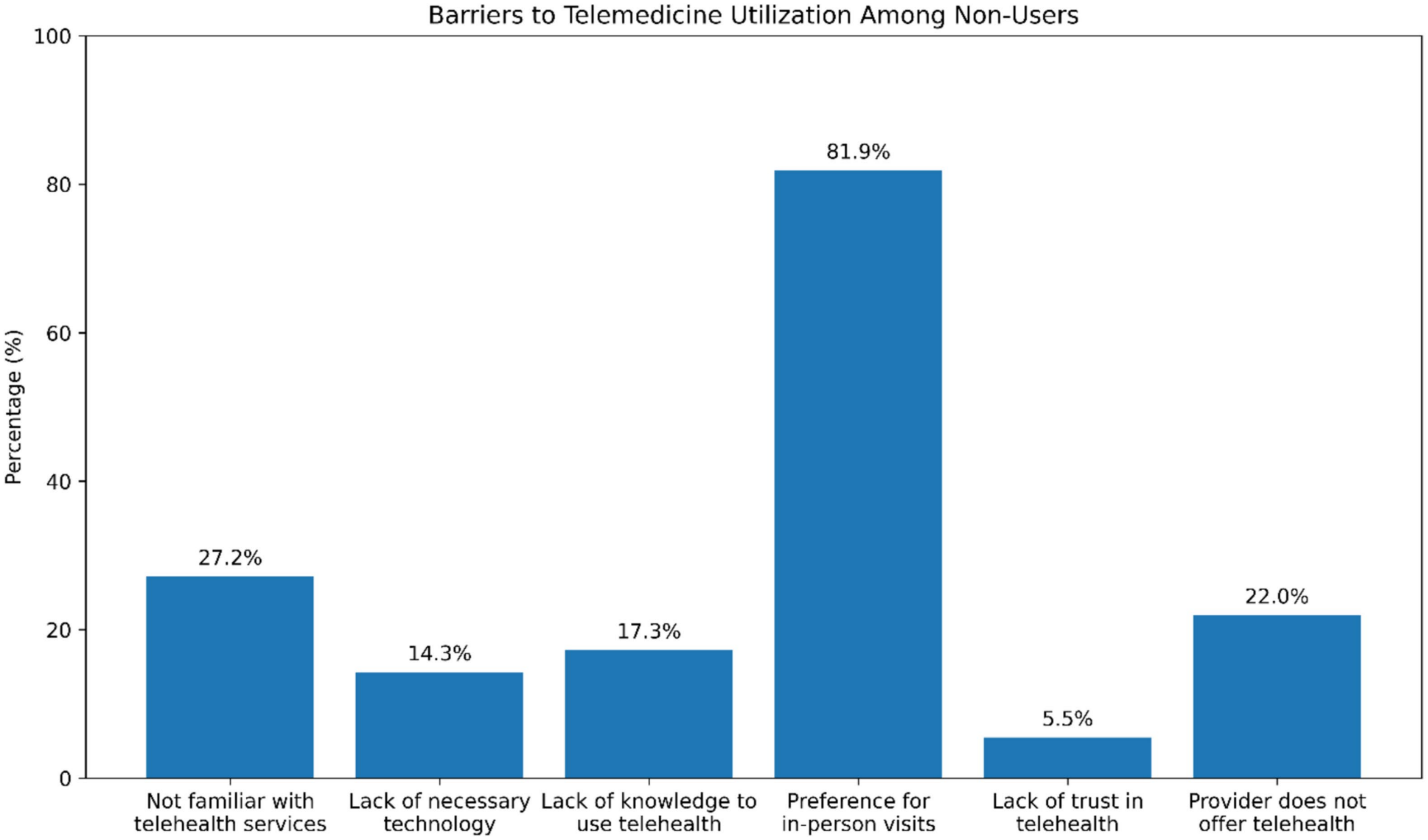
Barriers to telemedicine utilization among participants without prior telemedicine experience. The most commonly reported barrier was a preference for in-person healthcare visits (81.9%), followed by unfamiliarity with telehealth services (27.2%), and the absence of telemedicine services offered by healthcare providers (22.0%). Lack of technology (14.3%) and insufficient knowledge of telehealth platforms (17.3%) were less frequently reported, while distrust in telehealth services was minimal (5.5%). 3.6. Multivariate logistic regression analysis of factors related to telemedicine utilization.

Figure 3 illustrates the main barriers to telemedicine utilization reported by participants who had no prior experience with telemedicine services. Approximately 27.2% of respondents indicated that they were not familiar with telehealth services, whereas 72.8% reported being familiar with them. A smaller proportion (14.3%) identified lack of necessary technology as a barrier to telemedicine use, while 17.3% reported insufficient knowledge regarding how to use telehealth platforms. The most commonly reported barrier was a preference for in-person healthcare visits, reported by 81.9% of participants. In contrast, only 5.5% of respondents indicated a lack of trust in telehealth services. Additionally, 22.0% of participants reported that their healthcare provider does not offer telemedicine services. Overall, these findings suggest that personal preferences and limited availability of telehealth services remain important barriers to telemedicine adoption (Figure 3).

Table 5 presents the results of the multivariate logistic regression analysis investigating factors related to telemedicine utilization (Yes/No). Initially, each factor was analyzed separately to determine the crude odds ratio (COR). Subsequently, a final model was developed that included all associated factors to compute the adjusted odds ratio (AOR). Education level was significantly correlated with the utilization of telemedicine. Individuals possessing a school or pre-university education (AOR = 2.26, 95% CI: 1.19–4.28, *p* = 0.01) and those holding a university degree (AOR = 2.78, 95% CI: 1.23–6.29, *p* = 0.01) exhibited a higher likelihood of utilizing telemedicine in comparison to individuals who were illiterate. Similarly, urban residents had a significantly higher likelihood of using telemedicine compared to those in rural areas (AOR = 1.94, 95% CI: 1.23–3.07, *p* = 0.01). Widowed participants were significantly less likely to use telemedicine compared to those who had never married (AOR = 0.31, 95% CI: 0.11–0.89, *p* = 0.03). Although hearing sensory impairment was significantly associated with telemedicine use in the unadjusted model (COR = 0.52, 95% CI: 0.28–0.96, *p* = 0.04), this association was no longer significant in the adjusted model (AOR = 0.54, 95% CI: 0.27–1.09, *p* = 0.09). Other variables, such as work status, age, marital status, physical or visual sensory impairment, living situation, gender, and WHODAS score, did not exhibit significant associations with telemedicine use in the final model (*p* > 0.05). The findings indicate that elevated education levels and urban residency are significant positive associated factors of telemedicine utilization, while being widowed correlates with a reduced likelihood of adopting telemedicine (Table 5).

**Table 5:** Multivariate analysis of sociodemographic and disability categories related to telemedicine utilization.

Factors	Categories	Sig.	COR	95% C. I. for COR		Sig.	AOR	95% C.I. for AOR	
				Lower	Upper			Lower	Upper
Work status	Paid work (Ref)								
	Unemployed for health reasons	0.13	0.36	0.10	1.34	0.19	0.38	0.09	1.59
	Student	0.52	0.79	0.38	1.64	0.09	0.36	0.11	1.18
	Retired	0.87	1.06	0.52	2.18	0.42	1.47	0.58	3.71
	Unemployed for other reasons	0.95	1.03	0.46	2.30	0.82	1.12	0.41	3.03
	Housewife	0.20	0.68	0.37	1.24	0.41	1.41	0.62	3.19
	Self-employment	0.10	0.27	0.06	1.26	0.27	0.40	0.08	2.07
Age category	Less than 18 (Ref)								
	18–24 years	0.51	1.41	0.51	3.92	0.94	1.04	0.34	3.22
	24–39 years	0.42	0.76	0.39	1.48	0.08	0.35	0.11	1.13
	40–65 years	0.81	0.93	0.50	1.73	0.45	0.63	0.19	2.09
	More than 65 years	0.18	0.59	0.28	1.27	0.58	0.70	0.19	2.50
Marital status	Never married (Ref)								
	Currently married	0.90	1.03	0.65	1.62	0.45	0.76	0.37	1.56
	Separated	0.67	0.82	0.33	2.06	0.66	0.77	0.24	2.48
	Divorced	0.10	0.18	0.02	1.37	0.10	0.17	0.02	1.44
	Widowed	0.00	0.30	0.13	0.67	0.03	0.31	0.11	0.89
Education	I do not read or write (Ref)								
	School or pre-university	0.01	2.06	1.24	3.42	0.01	2.26	1.19	4.28
	University	0.01	2.26	1.28	3.97	0.01	2.78	1.23	6.29
	Post-graduate	0.08	3.31	0.88	12.47	0.09	3.97	0.82	19.22
Physical	No(Ref)								
	Yes	0.10	0.70	0.45	1.08	0.11	0.61	0.33	1.13
Visual sensory	No (Ref)								
	Yes	0.62	1.12	0.72	1.74	0.46	0.80	0.44	1.45
Living situation	Alone (Ref)								
	With family	0.23	1.94	0.66	5.75	0.54	1.46	0.44	4.87
Residence	Rural (Ref)								
	Urban	0.00	2.25	1.47	3.44	0.01	1.94	1.23	3.07
Hearing sensory	No (Ref)								
	Yes	0.04	0.52	0.28	0.96	0.09	0.54	0.27	1.09
Gender	Male (Ref)								
	Female	0.96	0.99	0.64	1.54	0.84	1.06	0.58	1.95
WHODAS score		0.53	0.94	0.78	1.14	0.60	1.06	0.85	1.34

AOR, adjusted odds ratio; COR, crude odds ratio; Sig, significance level; C.I., confidence intervals of the odds ratio.

## 4 Discussion

The provision of telehealth services that are accessible to those with disabilities is equivalent to enabling access for one in six persons around the globe. After the COVID-19 epidemic, telehealth services were used in a way that had never been done before. On the other hand, many individuals with disabilities continue to be unable to access these services, which makes it more difficult for them to get equitable medical treatment and has a detrimental effect on their health. This research aimed to evaluate the prevalence of telemedicine utilization among individuals with disabilities, examine factors affecting its adoption, and identify significant barriers to its use. A low adoption rate of telemedicine services was highlighted by the findings of this survey, which show that only 25.4% of participants reported utilizing them. Education level, residency, and the type of disability all showed significant differences. Problems with mental health were the most strongly linked to using telemedicine (χ^2^ = 45.915, *p* < 0.001), followed by problems with physical health (*χ*^2^ = 5.358, *p* = 0.021). Other types of disability, such as visual, hearing, neurological, and emotional, did not show statistically significant relationships with telemedicine usage (*p* > 0.05).

The high prevalence of difficulties in standing for long periods (49.2% reported moderate to extreme difficulty) and getting around independently (43.9%), from the WHODAS 2.0 results, suggests that many individuals in this study likely struggle with transportation to healthcare facilities. These findings are consistent with the significant association between transportation barriers and healthcare access limitations (*χ*^2^ = 83.615, *p* < 0.001). Our findings align with previous research on disability-related functional limitations and their impact on healthcare access (22). WHODAS scores were numerically higher among non-users of telemedicine, although the difference was small and not statistically significant. This finding suggests that overall functional limitation, as measured by WHODAS 2.0, may not independently differentiate telemedicine users from non-users in this population. The lack of statistical and clinical significance indicates that barriers to telemedicine adoption are likely driven more by structural, technological, and service-related factors than by differences in functional severity alone. Trouble participating in social activities (29.3% reported extreme difficulty) was one of the most frequently reported functional difficulties, aligning with previous research indicating that social isolation is a major concern among individuals with disabilities (23, 24). The results indicated that 32.8% of participants experienced moderate to extreme difficulty in emotional wellbeing. The findings align with global studies documenting a high prevalence of emotional distress and anxiety among individuals with disabilities, particularly attributed to limited healthcare access and challenges in social participation (25). The findings indicate that improved digital accessibility, assistive technologies, and targeted telehealth services for individuals with mobility impairments may reduce access barriers. Furthermore, the integration of mental health services into telemedicine platforms may enhance support for individuals facing social isolation and emotional distress, thereby improving healthcare accessibility for those with disabilities.

Disparities in adoption rate were noted according to education level and urban versus rural residence, with higher education levels and urban residency significantly enhancing the likelihood of telemedicine utilization. When compared with previous studies, this percentage is notably lower than the 34.5% telemedicine utilization rate reported among individuals with disabilities in the United States during the second year following COVID-19 pandemic (26). Similarly, research conducted in India discovered that 28 percent of people with disabilities have used telemedicine. Urban residency was independently associated with higher telemedicine utilization, highlighting persistent rural–urban disparities in digital health access. In rural and remote areas, limited broadband coverage, unstable internet connectivity, and reduced access to digital devices may constrain the feasibility of telemedicine use (27). Lower levels of digital literacy further compound these challenges, particularly among individuals with disabilities who may require additional support to navigate digital platforms (27, 28).

Beyond individual-level barriers, structural factors also play a critical role. Healthcare facilities in rural settings may have limited capacity to offer telemedicine services due to infrastructure constraints, workforce shortages, or lower institutional prioritization of digital health initiatives. This interpretation is supported by our finding that a substantial proportion of participants reported that telemedicine was not offered by their healthcare provider, suggesting that availability—not willingness alone—shapes utilization patterns.

In the Saudi context, these disparities may reflect uneven implementation of digital health services across regions, despite national efforts to expand telemedicine under ongoing healthcare transformation initiatives. Addressing rural barriers therefore requires not only improving internet infrastructure and digital literacy, but also strengthening provider engagement and ensuring equitable deployment of telemedicine services across urban and rural healthcare systems.

The study also revealed that the main difficulties about telemedicine were socio-cultural preferences for in-person consultations, technical illiteracy, and cost (29). In the context of Saudi Arabia, earlier research carried out during the COVID-19 pandemic revealed greater adoption rates of telemedicine than the rates that were seen in our study (30, 31). For example, a statewide research showed that 45.2% of Saudi citizens used telemedicine services during the epidemic, with the major motivation being the desire to avoid being infected (31). However, most of the currently accessible study concentrates on data collected during the pandemic era. Consequently, this emphasizes a considerable vacuum in the data about the adoption of post-pandemic telemedicine among persons with disabilities and suggests that variables other than pandemic-related situations may impact the usage of telemedicine among this demographic.

A key distinction in our study is the influence of disability types on telemedicine utilization. The results show that mental health problems are the strongest associated factors of telemedicine use (*χ*^2^ = 45.915, *p* < 0.001). This is in line with what we have seen around the world, where mental health services are some of the most popular telehealth options. Conversely, individuals with neurological, sensory, and emotional conditions exhibited no significant associations with telemedicine utilization (*p* > 0.05). This contrasts with a study from the US, which reported the highest utilization among mobility-impaired individuals at 43.3% and the lowest among those with hearing impairments at 34.5% (26). The results show that high-income nations incorporate telemedicine better into physical disability treatment. This difference is due to healthcare infrastructure, legislative support, and specialist telemedicine services. Digital health regulations and insurance coverage improve telemedicine for mobility-impaired Americans (32). The telemedicine services for physical disabilities in Saudi Arabia may be insufficient.

The emergence of Psychological conditions as the strongest associated factors of telemedicine utilization in this study reflects several contextual and service-level factors rather than a purely statistical association. First, mental health services are particularly well suited to virtual delivery, as they rely primarily on verbal communication and longitudinal follow-up rather than physical examination. In recent years, tele-mental health services have expanded substantially in Saudi Arabia and globally, increasing their accessibility within national digital health platforms and routine care pathways (10, 33, 34).

Second, telemedicine may reduce perceived stigma associated with seeking mental health care by offering greater privacy and minimizing face-to-face interactions, which may be especially relevant in sociocultural contexts where mental health stigma persists. Previous studies have shown that remote mental health consultations can lower psychological and social barriers to care and improve help-seeking behavior among individuals reluctant to attend in-person services (33, 34).

Finally, individuals with psychological conditions often require more frequent and ongoing follow-up, which may increase their exposure to and familiarity with telemedicine services over time. This pattern suggests that the observed association reflects the structural availability, acceptability, and continuity of tele-mental health services rather than inherent differences in digital readiness across disability groups (26, 32).

Security and privacy topped the TUQ-21 scale. Previous research has stressed the necessity of trust in digital health platforms for broad adoption (10, 33, 34). Recent cybersecurity developments, encrypted telemedicine platforms, and Saudi government data protection rules may explain our study’s strong security and privacy trust (9, 11). Telemedicine was easy to use and learn, according to participants. This aligns with global trends suggesting that user-friendly interfaces enhance the adoption of telemedicine (27). Security and favorable usability and learnability perceptions may not always make people more likely to use telemedicine because of problems with digital literacy and accessibility. Effective training and disabled-friendly interfaces might increase adoption by filling these gaps. Telemedicine was rated positively overall, but satisfaction and reliability scored slightly lower than security and usability, suggesting that while functional, some participants may have had consultation quality, interaction, or effectiveness issues. Previously, Low and Middle Income Countries (LMIC) research indicated that telemedicine satisfaction is worse in locations with intermittent internet access and poor healthcare provider engagement (28, 35). This may explain why some survey participants ranked satisfaction and dependability lower than security or simplicity of use.

Preference for in-person visits was cited as the most frequent barrier among those who do not use telemedicine, consistent with earlier studies showing that patients prefer face-to-face encounters for communication, trust, and treatment quality (10, 33, 34). In the Saudi Arabian setting, societal expectations about direct physician-patient connection may contribute to this considerable desire for in-person care (34). A recent research study on digital healthcare adoption in the Middle East found that patients often see physical consultations as more beneficial than virtual treatment, especially for complicated diseases that need hands-on examinations (36). A significant proportion of participants said that their healthcare provider did not provide telemedicine services, indicating structural constraints within the healthcare system. Similarly, results from other low- and middle-income countries (LMICs), indicated that adoption of telemedicine is frequently impeded by insufficient provider participation and infrastructural constraints (28). In contrast, high-income countries with well-established telehealth systems, such as Sweden and Canada, have reported greater provider engagement in telemedicine.

Notwithstanding the increasing worldwide acceptance of telemedicine, 27.2% of research participants indicated unfamiliarity with telehealth services, while 17.3% were unaware of how to use telemedicine platforms. The results align with a study conducted in India, which revealed that participants were unfamiliar with telemedicine, and had insufficient technical capabilities for its successful use (29). The obstacles with technology availability (14.3%) indicate that a modest but notable percentage of persons have difficulties in obtaining the essential gadgets and internet connection needed for telemedicine. Research conducted in rural Brazil identified low internet access and insufficient digital devices as major impediments to the spread of telehealth in underserved regions (37). In contrast to other research highlighting security and data privacy issues as significant obstacles to telemedicine, our results reveal that just 5.5% of participants showed a lack of confidence in telehealth services. This suggests that trust is not a big issue for this group of people when it comes to using telemedicine. This may be because of better security measures and laws that protect patients’ privacy.

### 4.1 Strengths and limitations

This research exhibits multiple strengths. The study utilizes validated assessment tools, WHODAS 2.0 and TUQ-21, to apply standardized measures for evaluating functional limitations and user perceptions of telemedicine. A multivariate logistic regression model was employed to control for potential confounding factors, thereby clarifying the independent effects of associated variables on telemedicine utilization. The comparative approach enhances the study by contextualizing findings across high-income and low- and middle-income countries, providing a broader understanding of factors influencing telemedicine adoption. Importantly, this study addresses a significant gap by examining telemedicine utilization among individuals with disabilities in the post-pandemic period, whereas much of the existing literature focuses primarily on pandemic-era trends.

Nonetheless, several limitations should be acknowledged. Facility-based recruitment may have introduced selection bias by preferentially capturing health-service–engaged individuals with disabilities while underrepresenting those who rely exclusively on remote care or are disengaged from in-person services, potentially affecting generalizability. Data were collected through face-to-face interviews, which may have introduced social desirability and interviewer-related bias, particularly in sensitive domains such as mental health, emotional distress, and healthcare-seeking behaviors. However, this approach was necessary to facilitate participation of individuals with sensory, communication, or functional impairments who may have difficulty completing self-administered or online questionnaires.

The study population was also heterogeneous, including adults with diverse disability types as well as parents of children with disabilities, which may have introduced variability that could not be fully explored through stratified analyses. Psychological conditions showed the strongest association with telemedicine use; however, the study cannot determine whether this reflects increased availability of tele-mental health services, reduced stigma, or other contextual factors. Additionally, while urban residency was associated with higher telemedicine utilization, the study did not directly assess rural-specific barriers such as internet connectivity or digital literacy. The finding that 22% of participants reported that telemedicine was not offered by their healthcare providers suggests structural barriers within the healthcare system, although institutional policies related to telemedicine implementation were not evaluated. Finally, the cross-sectional design precludes assessment of temporal changes in telemedicine use. Longitudinal studies are needed to evaluate how digital health interventions and policy reforms influence sustained adoption among people with disabilities.

### 4.2 Policy implications

Based on the identified barriers and facilitators, several system-level actions are warranted to support equitable telemedicine adoption among people with disabilities in Saudi Arabia. First, telemedicine availability should be standardized across primary, secondary, and tertiary care, with minimum service benchmarks and accountability indicators to reduce provider-level variation. Given that nearly one-quarter of participants reported that telemedicine was not offered by their healthcare providers, institutional policies should mandate telemedicine integration within routine outpatient services, supported by workforce training and performance monitoring.

Second, targeted digital inclusion strategies are needed for rural residents and individuals with lower educational attainment, including assisted telemedicine onboarding, simplified user interfaces, and community-based digital literacy programs embedded within primary healthcare and rehabilitation settings. Third, disability-inclusive platform standards—such as screen-reader compatibility, captioning, and alternative communication options—should be formally incorporated into national digital health procurement frameworks to ensure accessibility for users with sensory and functional impairments.

The higher utilization observed among individuals with psychological conditions supports expansion of structured tele-mental health pathways, with clearer referral integration between primary care, rehabilitation services, and mental health providers. Finally, hybrid care models combining in-person and virtual visits may help address persistent preferences for face-to-face care while preserving the convenience of remote services. Collectively, these measures align with Saudi Vision 2030 health transformation objectives and provide actionable pathways to reduce digital health inequities among people with disabilities.

## 5 Conclusion

This study demonstrates a persistently low level of telemedicine adoption among people with disabilities in Saudi Arabia in the post-pandemic period, with clear disparities associated with educational attainment, place of residence, and type of disability. Psychological conditions showed the strongest association of telemedicine use, reflecting the relative suitability, availability, and acceptability of tele-mental health services compared with other forms of specialized care. Notably, favorable perceptions of security, privacy, and convenience were insufficient to translate into widespread utilization, underscoring the importance of structural and system-level determinants of access.

The findings indicate that barriers to telemedicine adoption are driven less by user trust and more by a combination of strong preferences for in-person care, inconsistent availability of telemedicine at the provider level, and gaps in digital literacy. To align with Saudi Arabia’s Vision 2030 and ongoing health sector transformation, policy efforts should prioritize standardizing telemedicine provision across healthcare facilities, particularly within primary and rehabilitation care. Targeted digital inclusion strategies—such as assisted telemedicine onboarding, simplified platforms, and community-based digital literacy support—are especially needed for rural residents and individuals with lower educational attainment. In parallel, the high uptake among individuals with psychological conditions supports further expansion of structured tele-mental health pathways integrated into routine care.

Given the limited post-pandemic evidence on telemedicine use among people with disabilities, future research should focus on longitudinal adoption patterns, provider-level implementation practices, and the impact of national digital health policies on equitable access. Addressing these priorities through coordinated regulatory action, workforce engagement, and inclusive technology design is essential to realizing the full potential of telemedicine in reducing healthcare disparities for people with disabilities in Saudi Arabia.

## Data Availability

The original contributions presented in the study are included in the article/supplementary material, further inquiries can be directed to the corresponding author.
